# Immune checkpoint inhibitor (ICI) genes and aging in malignant melanoma patients: a clinicogenomic TCGA study

**DOI:** 10.1186/s12885-022-09860-2

**Published:** 2022-09-13

**Authors:** Mohammed Safi, Chenxing Jin, Abdullah Aldanakh, Ping Feng, Henan Qin, Mohammed Alradhi, Lizhi Zhang, Junying Zhang, Salah Adlat, Yi Zhao, Jiwei Liu

**Affiliations:** 1 Department of Oncology, First Affiliated Hospital of Dalian Medical University, Dalian, 116021 China; 2 Department of Urology, The First Affiliated Hospital of Dalian Medical University, Dalian, 116021 China; 3 Department of Urology, The Affiliated Hospital of Qingdao Binhai University, Qingdao, China; 4 Department of Pathology, First Affiliated Hospital of Dalian Medical University, Dalian, 116021 China; 5 Department of Gastroenterology, Department of Medicine, Perelman School of Medicine Philadelphia, University of Pennsylvania, Philadelphia, PA USA

**Keywords:** Aging, Immune checkpoint inhibitors (ICI), TNFRSF4, Malignant melanoma, Survival epidemiology

## Abstract

**Background:**

Cancer diagnoses and deaths among the elderly (65 +) are expected to increase significantly over the next decade. Immune checkpoint inhibitors specifically target ICI genes and enhance immune system function. However, poor outcomes may be associated with aging.

**Methods:**

We downloaded the Genomic Data Commons from the Cancer Genome Atlas (TCGA) and collected gene expression data from malignant melanoma (MM) tissues, the third level as the primary site. The CKTTD ICI genes database were applied and validated using the GEO database and lab experiments.

**Results:**

In 414 patients, 13 ICI genes were obtained as risk gene signature by univariate and multivariate Cox hazard models and were associated with poor survival in the older group. At 1, 3, and 5 years (79%, 76%, and 76%, respectively), we investigate TNFRFS4 gene and age prediction using novel nomogram-associated aging (HR = 1.79, P 0.001, CI = 1.32–2.45) with higher sensitivity testing.TNFRSF4 gene expression was significantly high in younger (15 years interval) MM patients (*P* < 0.001). By correlation analysis, a significant negative association was determined (*P* < 0.001). The validation of gene correlation from GEO (GSE59455) and (GSE22153) was obtained as external validation. We tested the TNFRSF4 protein levels by IHC in 14 melanoma tissue samples. TNFRSF4 expression was observed to be lower expressed in the older of melanoma tissues, and higher in the younger age group (*P* = 0.02). Besides the connectivity of ICI gene proteins, the biological processes of cell aging, aging, and the immune system were found to be highly related.

**Conclusions:**

Along with the risk score evaluation, the ICI gene (TNFRSF4) was identified as a tumor suppressor gene related to inequalities in age survival and associated with immune cell infiltrations. The aging responses of melanoma patients and related gene expression need further investigation in order to identify potential therapeutic targets.

**Supplementary Information:**

The online version contains supplementary material available at 10.1186/s12885-022-09860-2.

## Introduction

It is critical to have a high degree of tolerance to chemotherapy treatments. As a result of the development of breakthrough immunomodulatory antibody therapies that depend on the patient's immune system to manage malignancies in recent years, the issue of immunosenescence has risen to prominence on the medical agenda, prompting a flurry of new research and discussion [1–3]. The discovery of immune checkpoint inhibitors, which target the programmed cell death 1(PD-1), programmed cell death ligand (PDL1), and cytotoxic T lymphocyte antigen 4 pathways, has resulted in the development of new cancer therapies [4, 5]. This method, which involves targeting the immune system, has shown remarkable efficacy in the treatment of many cancers, and several medications have been licensed by health authorities and are now in clinical trials [6].

Patients over the age of 65 account for the vast majority of cancer diagnoses and fatalities, and it is expected that this age group will grow significantly over the next ten years. However, this particular subset of patients is underrepresented in clinical studies. Furthermore, aging is associated with a decline in the immune system's effectiveness and alterations in the immune system. Although most patients are considered healthy, there has been little research, particularly on immunotherapy in the elderly [7]. Anecdotal evidence shows that older patients getting anti-CTLA-4 or anti-PD-1/PDL1 antibodies do not have lower responses or higher toxicity due to their advanced age, which is a promising development [8]. This may be explained by the fact that the link between frailty in older persons and distinct T-cell subset "profiles," which may have consequences for determining how to effectively assess older patients with varying basic functional status in terms of the degree of responsiveness, safety, and overall therapeutic effects of immunotherapy within an immunosenescent setting [9]. Because of the enormous amount of research showing that practically all measures of innate and adaptive immunity change between younger and older people, the true clinical importance of these variations is still unclear, and assessing immunosenescence status using markers is a challenging task [10]. While recognizing that the majority of published studies are cross-sectional in nature (and thus may detect only differences rather than changes), the vast majority of them provide no evidence that observed age-related disparities in the immunological parameters examined are harmful to the individual.The first ICI drug was approved for MM patients, considerably improving survival compared to non-users, and the research of genomic expression changes with aging by using public datasets TCGA and wet experiments is becoming increasingly important to be investigated.

Our study will screen the genetic level of MM patients by using the TCGA database. Using the most recent selected ICI gene list (CKTTD) and matching it with transcriptomic expression data from the TCGA, we will investigate the molecular profiling of MM patients and find age-related ICI gene expression. In addition to the experimental lab work, the derived age-related gene was validated from the GEO database.

## Materials and methods

### Data sources and study parameters

We downloaded the Genomic Data Commons (GDC, accessible via the portal: https://portal.gdc.cancer.gov/) and managed to collect gene expression data from MM tissues at the third level. For tumors with "MM" as the main site, the expression profiles for MM projects contained profiles for tumors with the disease forms of "cutaneous skin melanoma". Patient ID, gender, age, stage, and survival information were also retrieved from the database for the corresponding patients. Cancer patients with survival and expression data available when the research was done were the only participants in this report. The total number of protein-coding genes that have been annotated in the TCGA data portal that used fragments per kilobase of exon per million mapped (FPKM) as a measurement was 19,658. Access to the de-identified linked dataset was obtained from TCGA to analyze identified data from the TCGA. The resulted coding genes were matched with new recent CKTTD ICI genes [11]. Institutional review board approval and informed consent were not required.

### Construction of a prognostic gene signature

The Cox hazard model by univariate and multivariate was used to compare ICI expression levels and the expression of prognostic genes in the high- and low-risk groups. MM patients were divided into high-risk and low-risk groups according to the median value of the risk score, and the OS of patients were analyzed by the Kaplan–Meier (KM) method and log-rank test. The gene and age correlations were applied to the highly significant results from the independent test results.

Gene ontology (GO) and Kyoto Encyclopedia of Genes and Genomes (KEGG) and construction of the PPI network and immune cell infiltration. GO and KEGG searches were carried out using the websites https://biit.cs.ut.ee/gprofiler/gost [12–15]. We obtained the protein–protein interaction (PPI) network of ICI genes using Cytoscape software (https://cytoscape.org/) to reconstruct and visualize the network of the ICI gene database. Estimated immune cell infiltration was performed by the R package according to the risk score.

### Statistical analysis

We first obtained the prognostic genes in MM by using univariate Cox hazard analysis. We performed multivariate Cox hazard analysis using the survival package in R. Independent tests, one-way ANOVA on ranks, and correlation analysis were also applied. We considered a P value of less than 0.05 and a limit of 0.001 as statistical significance. All statistical methods are done using the R program version 4.0.4 and SPSS version 25.

### Experimental methods Immunohistochemistry (IHC)

We used the hematoxylin and eosin (H&E) stain, a well-established method for detecting gene expression in tissue, to check for differences between normal and tumor tissue and study the difference between different age groups.14 human melanoma tissues were provided (2018–2020) by Dalian Medical University's First Affiliated Hospital in accordance with Dalian's ethics committee for human research and the patient’s written consent was taken.

### Formalin-fixed, paraffin-embedded tissue sections

The newly dissected tissue (about 3 mm thick) was preserved with 2% paraformaldehyde at room temperature overnight. Afterwards, the tissue was washed with running tap water for 5 min before being dehydrated with 70% alcohol, 80% alcohol, and 95% alcohol for 5 min each, followed by three times with 100% alcohol for 5 min each. The tissue was cleared in xylene twice for five minutes each time and then immersed in paraffin three times for five minutes each time. The paraffin-embedded tissue was sectioned on a microtome at 5–8 m thickness and floated in a 40 °C water bath containing distilled water. We transferred the sections onto glass slides suited for IHC, allowing the slides to dry overnight before storing them at room temperature until ready for use.

### Immunostained sections of formalin-fixed, paraffin-embedded tissue

After the paraffin sections were melted in the oven at 65˚C for 60 min, the tissue slices with a thickness of 3 to 5 microns were cooled at room temperature. Following deparaffinization in xylene for 10 min in two changes, the tissues were rehydrated with serial ethanol dilutions for 5 min in absolute ethanol concentrations of 95%, 85%, 75%, and 50%. The tissues were washed with water for 3 min and washed three times in PBS for 5 min each time. The heat induced epitope retrieval method was used, and the samples were incubated with a 3% hydrogen peroxide solution for 10 min before being washed for 5 min and three times with PBS. After removing the blocking buffer from the slides, primary antibodies were added and incubated at room temperature for 1 h (TNFRSF4; 1:100-Huabio). Tissues were rinsed three times with PBS, then incubated for 15 min at room temperature with HRP-conjugated secondary antibody and washed three times for three minutes each time. After 10 min of exposure to DAB stain, stained antibodies were washed three times with PBS to eliminate any left-over staining. After staining with H & E for 3–5 min, all slides were rinsed in water and immersed in serial ethanol dilutions of 50%, 75%, 85%, %, 95%, and 100% for 3 min each. The slides were finally counted by using a cover slip on the tissue using DPX adhesive and waited for it to dry. According to the number of chromogenic and the color depth of cells, the score from two pathologists inspected the slides independently under a 20 × magnification light microscope. Then we used the average from the pathologists to make the results.

## Results

### Data analysis and constructing prognostic gene signature

Following the selection of the only known and available data on MM, 414 patients were extracted from the TCGA database, which is shown in Supplementary Table 1. When the transcriptomic gene expression was matched by the ICI genes (CKTTD), the univariate hazard model regression analysis was performed, which indicated only 40 genes with a statistically significant difference in low vs high expression (considering only *P* value = 0.05); (CD27,VSIR,ICOS,HAVCR2,IDO1,CD80,CTLA4,TNFRSF18,TIGIT,TNFRSF4,CD274,TNFRSF9,PDCD1,ISYNA1,CSF1,CD47,TLR8,ARG1,TREM2BMI1,TNFRSF1B,BTK,LILRB1,PLK1,CD52,CCR5,TLR3,TLR7,CREBRF,TLR9,TLR4,LILR2,CXCR4,CCR2,NOX4,CD96,CD38,AXL,NR2F6). (Supplemetary1 A). Further multivariate hazard model analysis revealed 13 ICI genes which were supposed to be as risk gene signature (ICOS, CD80,CTLA4,TNFRSF4,PDCD1,CSF1,TLR8, ARG1,BMI1,TLR3,TLR4,NOX4, AXL) and the top significant 6 genes was selected as risk score genes (considered only *P* value < 0.05) (ICOS, CD80, NOX4,ARG1,TNFRSF4, PDCD1) (Supplementary Fig. 1).

The KM survival analysis was undertaken to evaluate overall survival (OS) results to identify the potential of each ICI gene signature-related OS to predict the prognosis of MM patients. The overall survival rate for patients in the high-risk group was considerably lower than the rate in the low-risk group in the risk score. (log-rank *P*-value < 0.0001) (Supplementary Fig. 2 A).

**Fig. 1:**
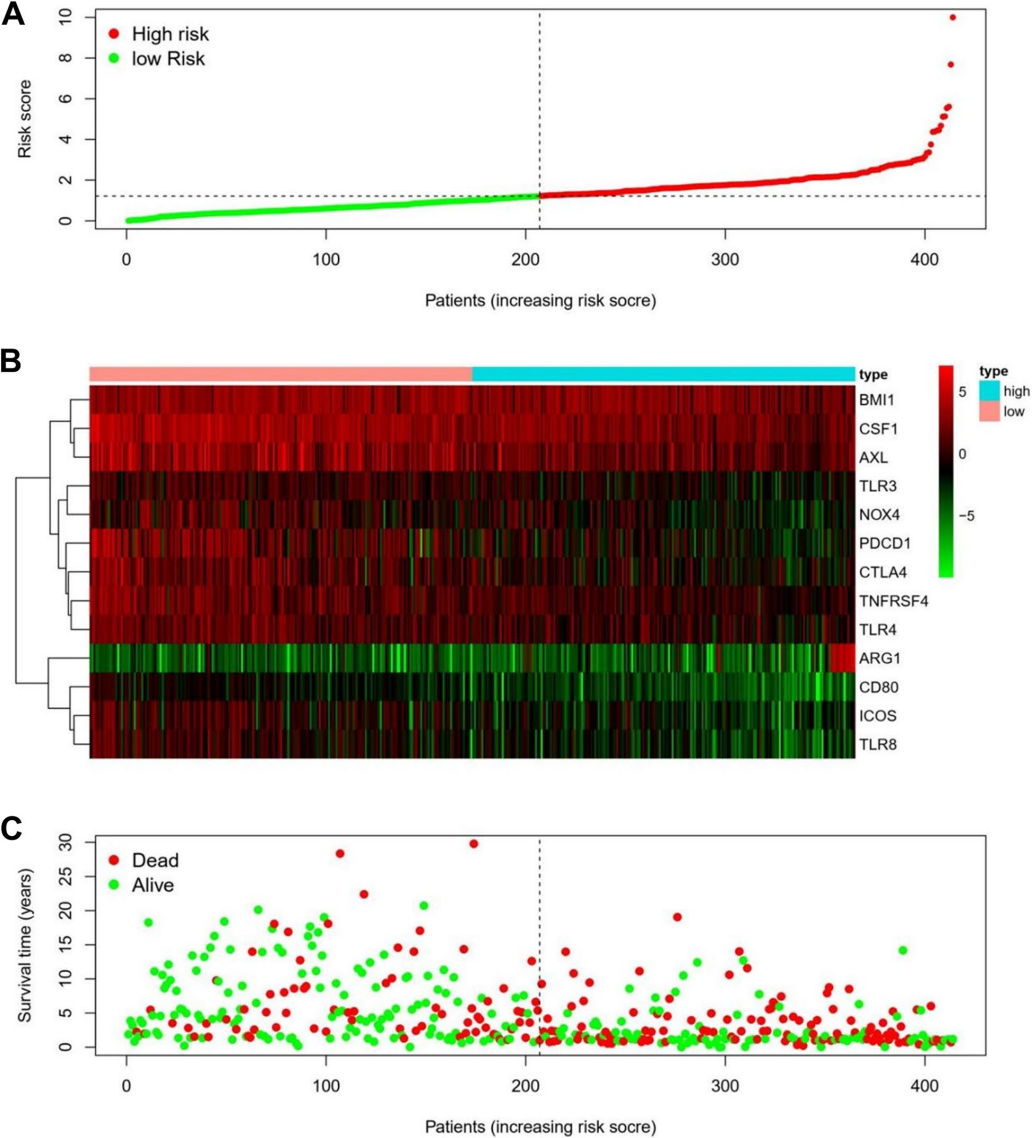
Risk score distribution of the gene signature of patients among OS cohort. represent the gene signature heatmap; Red lines indicate low-risk group and blue lines for a high-risk group, respectively

This study evaluated the 6-gene signature's predictive potential using a nomogram (Supplementary Fig. 2B). Thus, patients' risk scores are divided into two groups: those at great risk and those who are not; the higher a patient's risk score, the greater the possibility of death (Fig. 1). It was determined that all of the risk score genes are oncosuppressors (their high expression was related to greater survival than their low expression). (*P* < 0.05) (Supplementary Fig. 3).

### Ageing-related genes

**Fig. 2:**
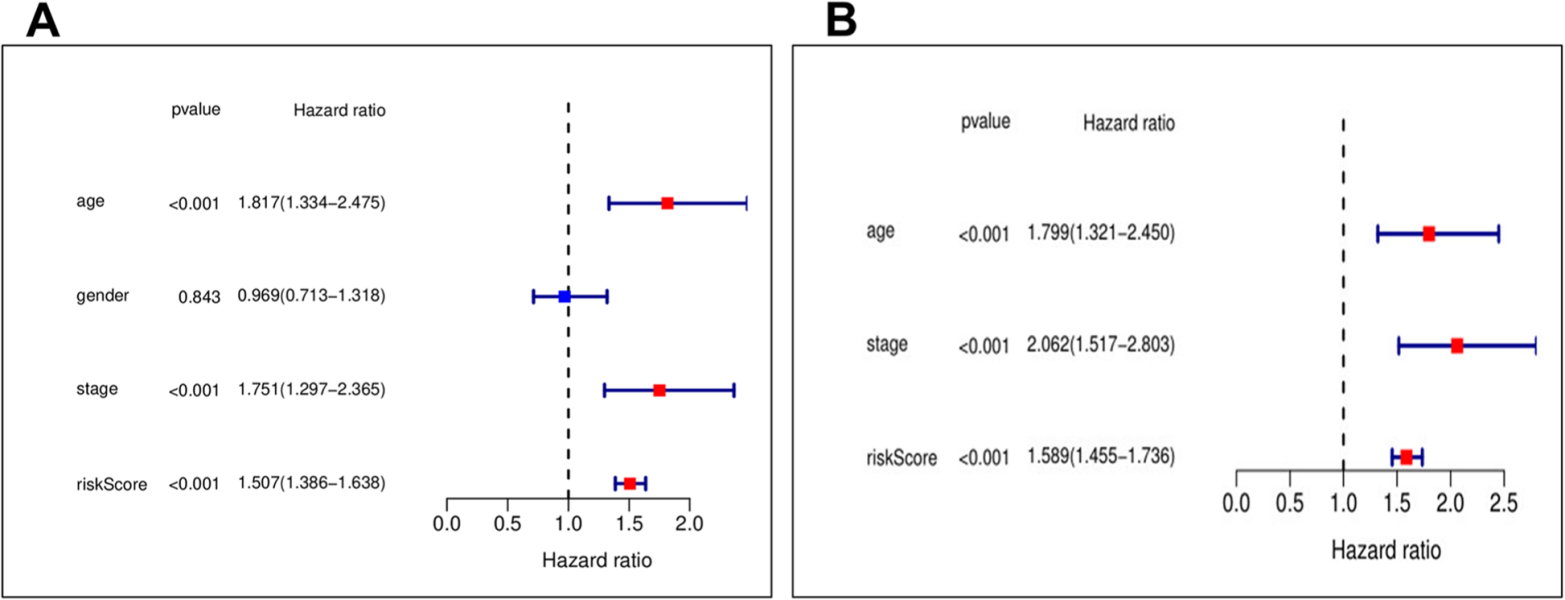
Prognosis discovered by univariate and multivariate survival analysis for clinical factors associated with a risk score. The age variable independent risk variable significant (HR = 1.79, *P* =  < 0.001, CI = 1.32- 2.45)

An effective strategy for predicting prognosis was discovered after a multivariate cox hazard model analysis for clinical factors associated with a risk score. In addition to the risk score as the independent variable (HR = 1.58, *P* < 0.001, CI = 1.45–1.73), the age variable was found to be statistically significant (HR = 1.79, *P* < 0.001, CI = 1.32- 2.45) (Fig. 2).

**Fig. 3:**
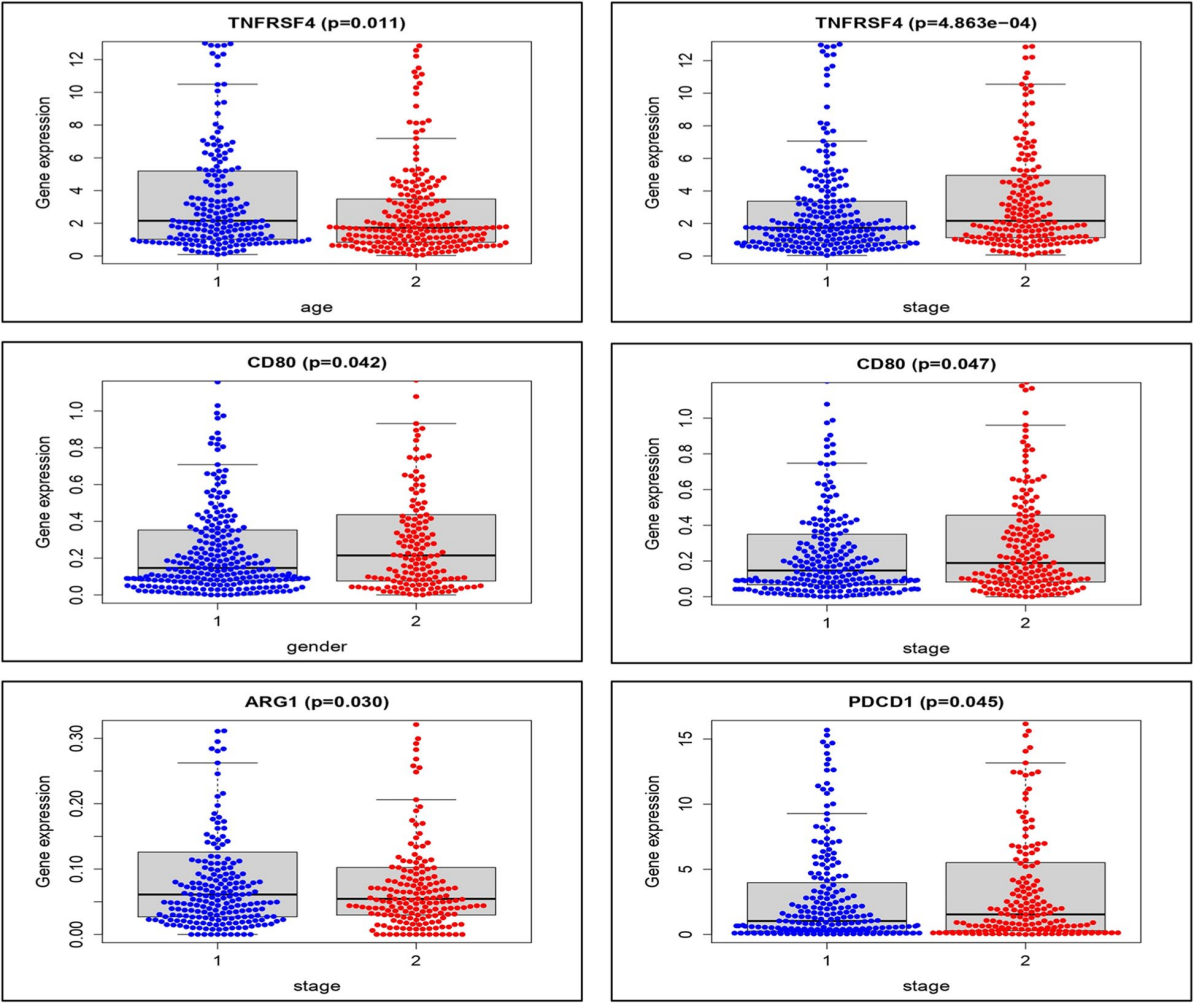
The median expression association with relevant clinical factors. TNFRSF4 gene expression was shown to be significantly linked with aging in patients with malignant melanoma.(*P* = 0.01)

**Fig. 4:**
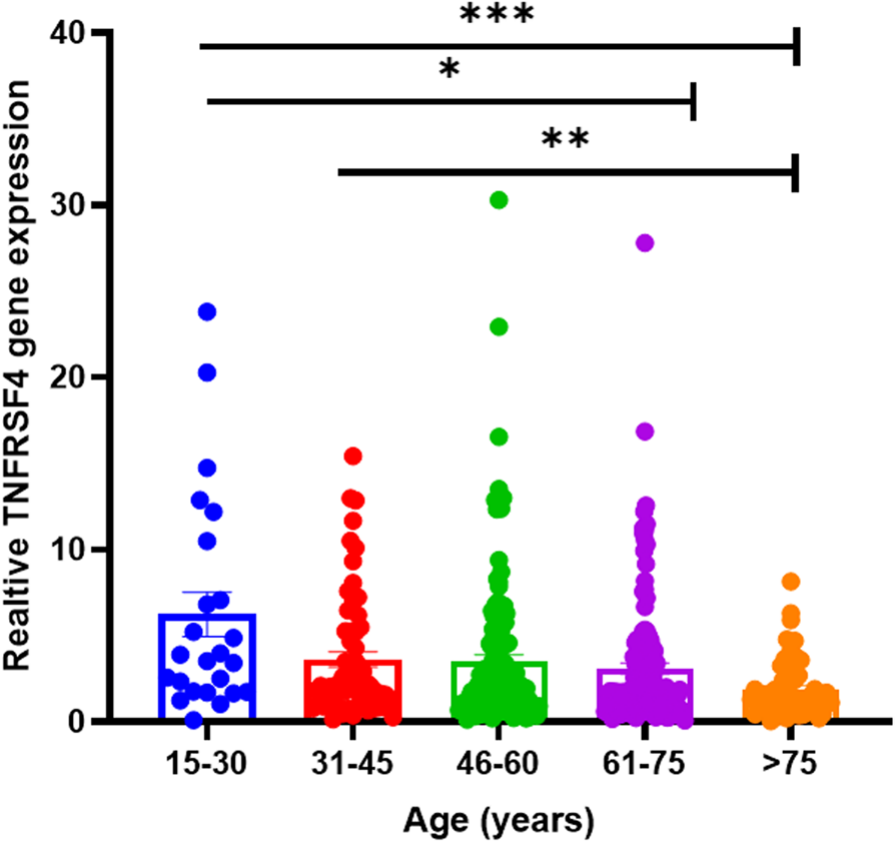
Significant difference between each age group with 15-year interval in training group

**Fig. 5:**
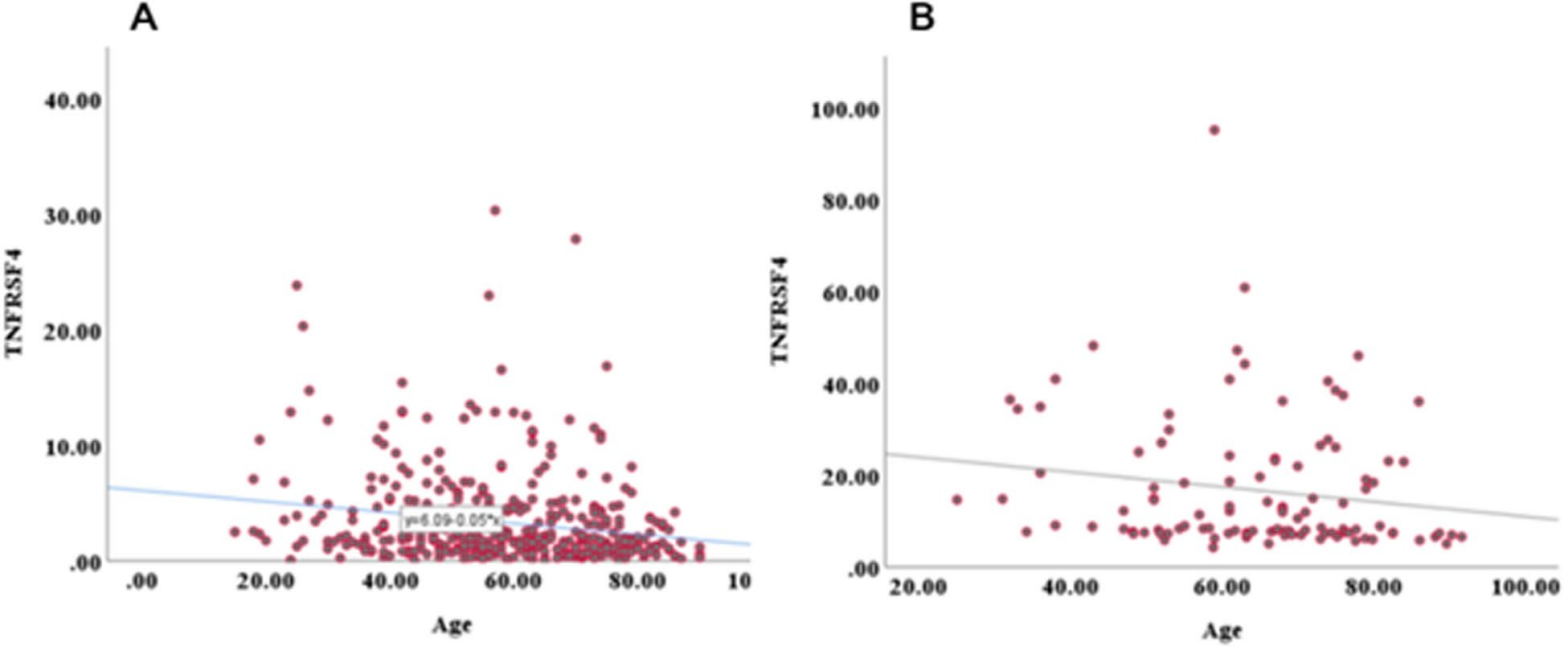
TNFRSF4 gene expression from TCGA and validation of gene correlation from GEO (GSE59455) and (GSE22153) as external validation determined (*P* =  < 0.001) with r = -0.20. (*P* = 0.01) and r = -0.24)

According to the independent test, the expression association with relevant clinical factors showed TNFRSF4 gene expression was significantly linked with aging in patients with MM (*P* = 0.01) (Fig. 3). The strong significance between younger age group and the eldest one by one-way ANOVA on ranks results was clearly seen (15-year interval) ( *P* < 0.001) (Fig. 4). In addition to the correlation analysis in the training group, validation of gene correlation from GEO (GSE59455) and (GSE22153) were obtained as external validation. ((*P* < 0.0001) and r = -0.20, *P* = 0.01, r = -0.24) respectively (Fig. 5).

**Fig. 6:**
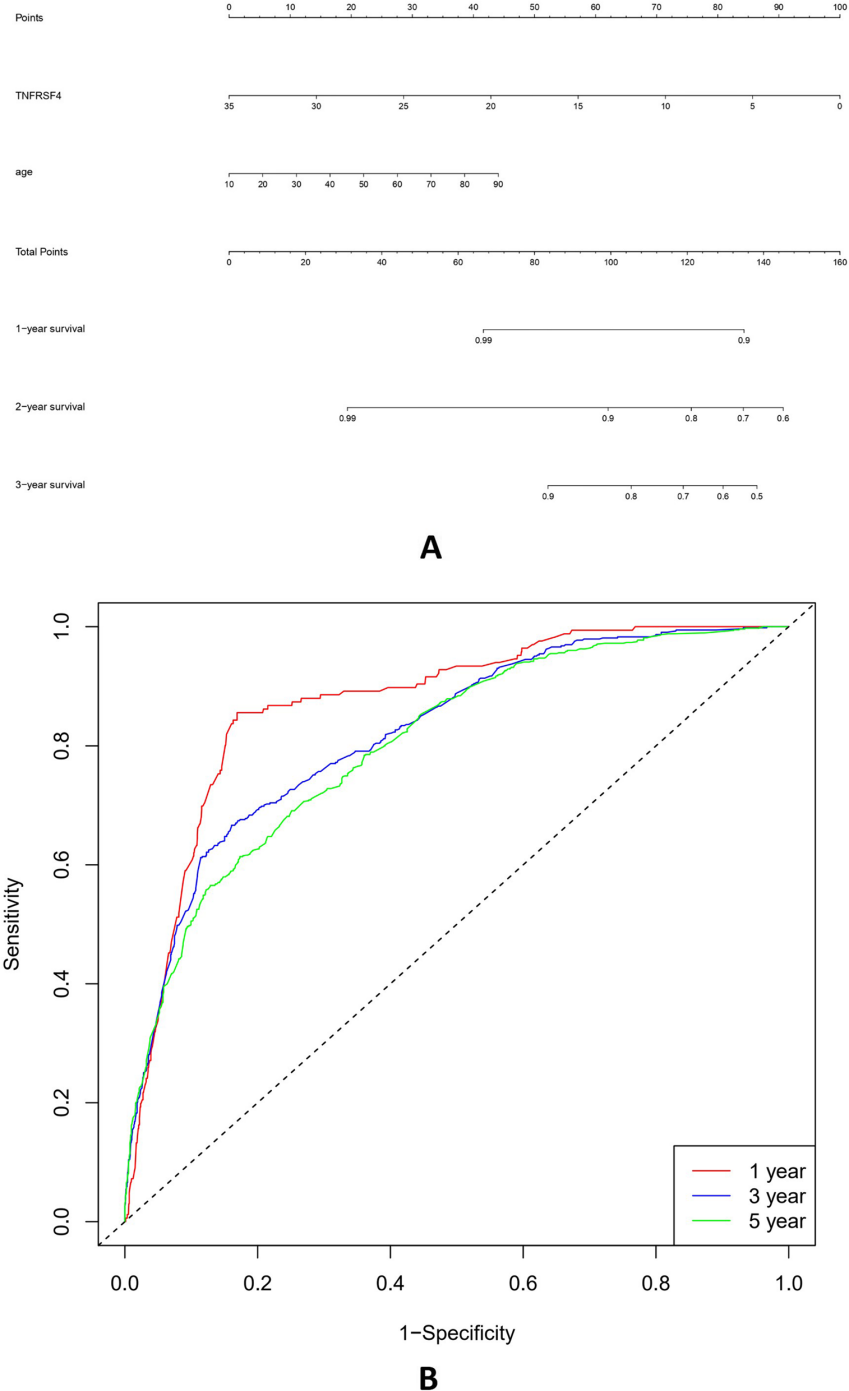
Prediction of TNFRSF4 gene by novel nomogram which was highly detecting the risk of death and associated age with higher sensitivity testing in one, three, and fifth-year AUC (79%, 76%, and 76%)

**Fig. 7:**
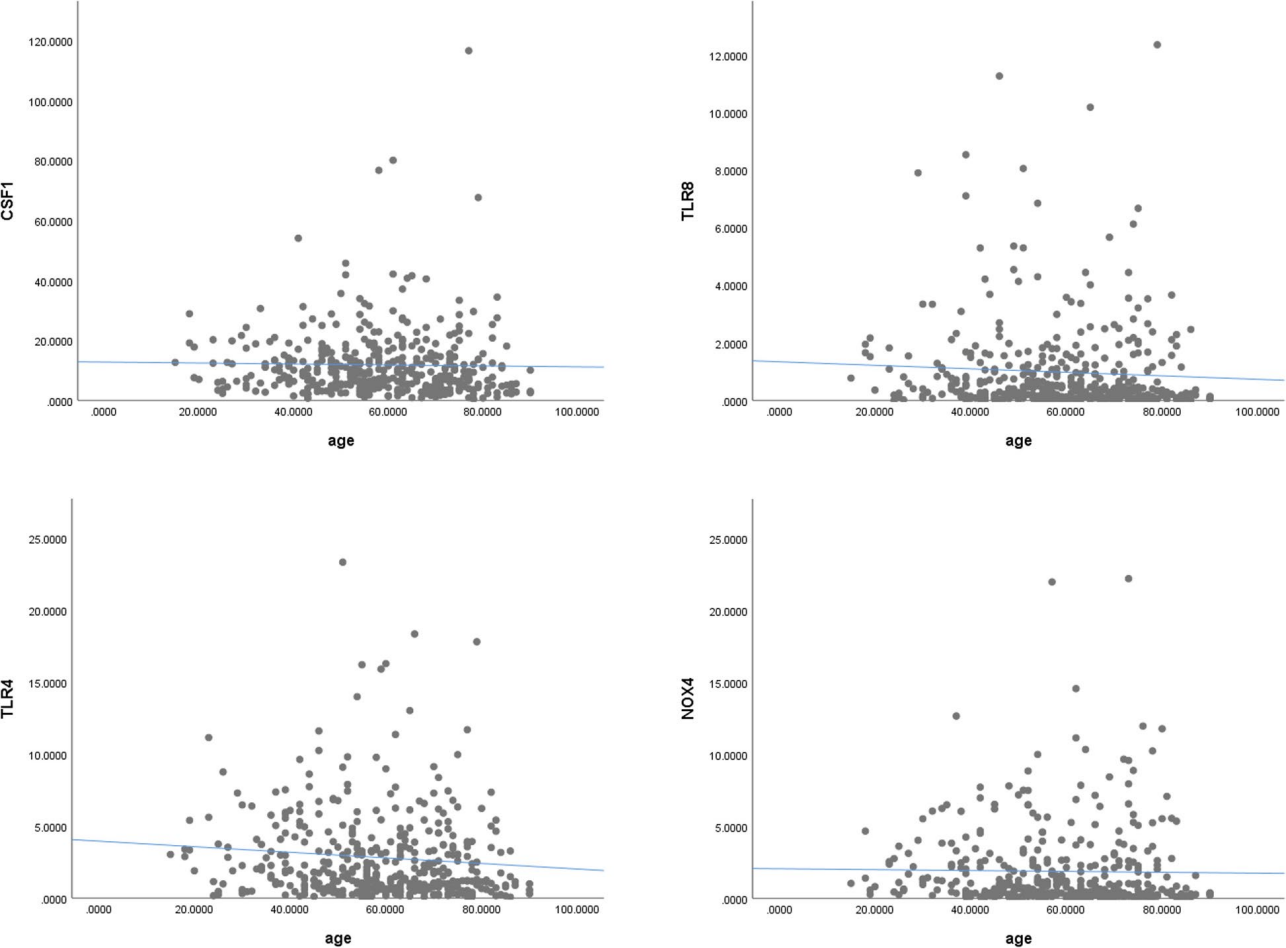
The correlation between risk score genes resulting from multicox and age showed negatively correlated with age (CSF1, TLR8, TLR4, NOX4) (*P* =  < 0.05)

We investigate the prediction of TNFRSF4 gene and age by novel nomogram that highly detects the risk of death and associated age with higher sensitivity testing in one, three, and fifth-year AUC (79%,76%,76%), respectively (Fig. 6). By examining the correlation between risk score genes resulting from multivariate cox hazard model and age, we discover four genes that were likewise shown to be negatively correlated with age (CSF1, TLR8, TLR4, NOX4) (P < 0.05) (Fig. 7).

### GO, KEGG, and construction of the PPI network

GO analysis revealed that genes associated with CKTTD were prominent in the biological processes of aging and cell aging with immune system. KEGG analysis revealed that pathways of cytokine-cytokine receptor interaction and cell adhesion molecules were associated (Supplementary Table 2). ICI genes were illustrated and described in the context of the PPI network for the CKTTD database (Supplementary Fig. 4).

### Immune cells infiltration

Immune cells are distributed among risk classes were selected the most significant positive correlation of the risk genes concerning the abundance of immune cells that measured by Timer (http://timer.cistrome.org) for infiltrative immune cells between high and low-risk groups. In addition, it allows visualizing the correlation of its expression with immune infiltration level in MM. The scatterplots generated and displayed Spearman’s rho value and statistical significance of the risk score gene signature by using R package (Supplementary Fig. 5).

### Experimental validation: The protein expression levels of TNFRSF4 using IHC

**Fig. 8:**
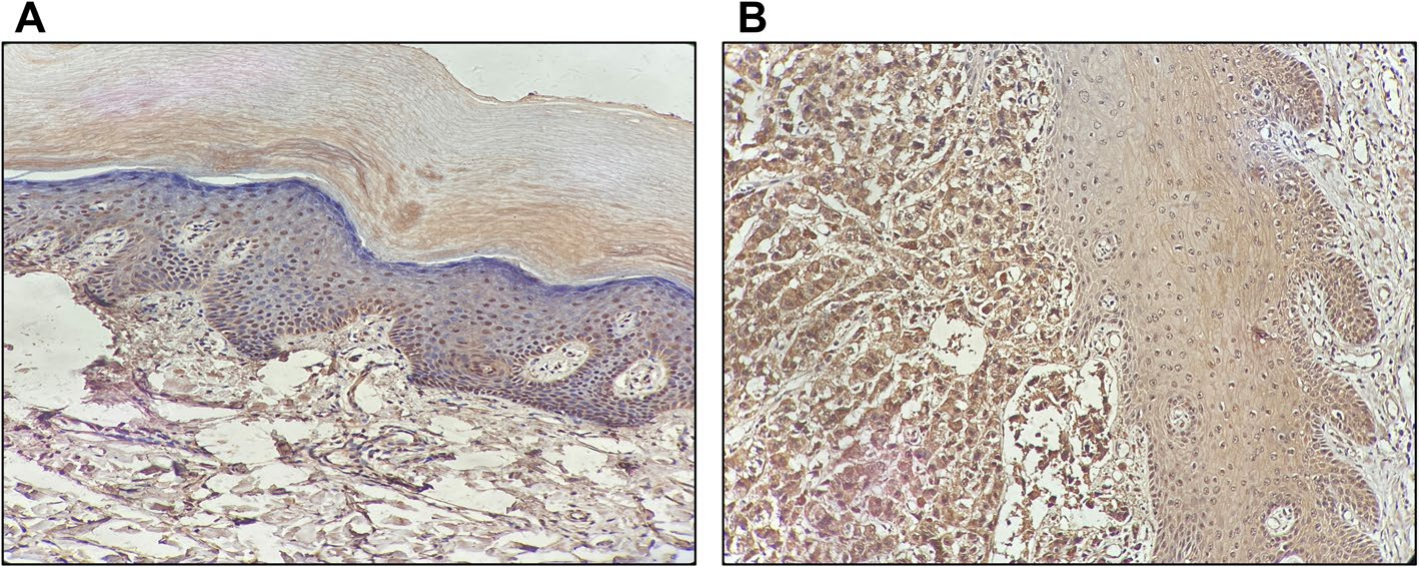
A. Skin tissue showing normal TNFRSF4 protein expression. B. Primary malignant tissue showing high chromogenic and depth of color cells

**Fig. 9:**
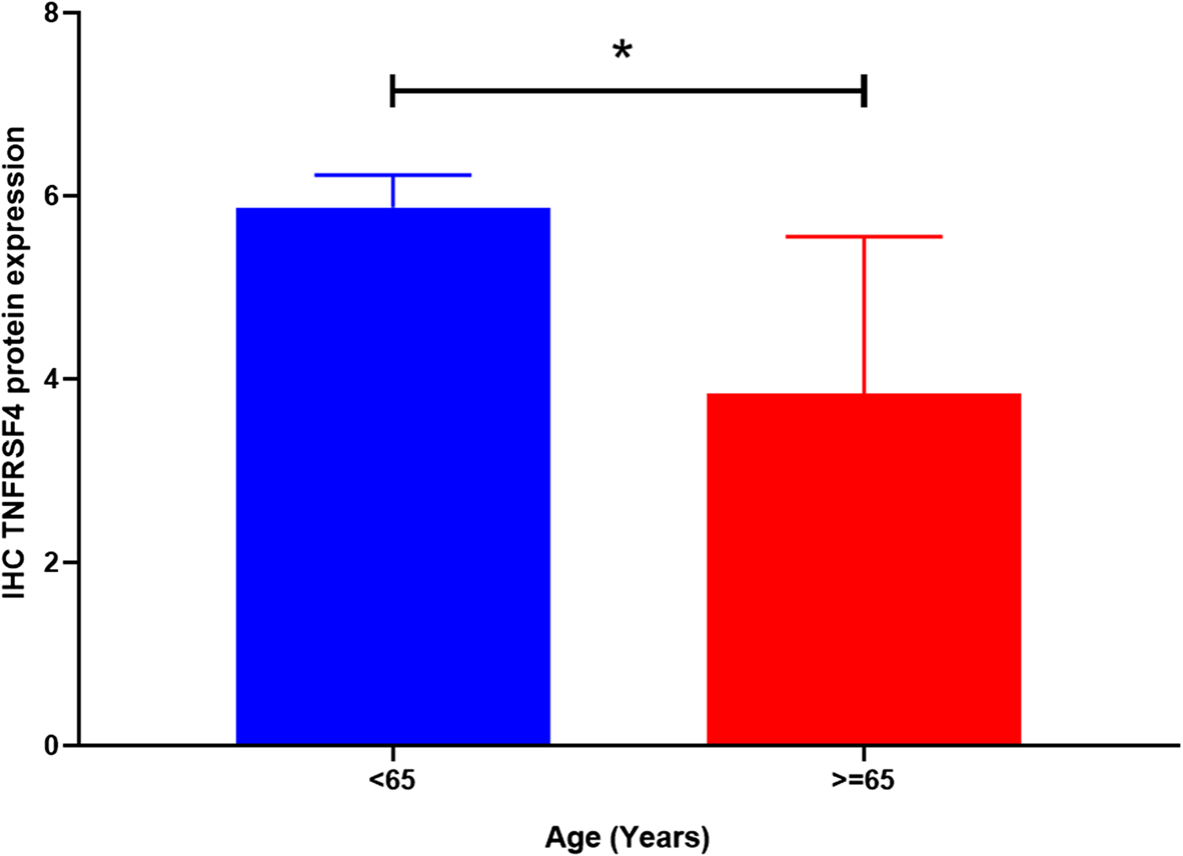
Immunohistochemistry of TNFRSF4 protein expression. The expression in the older group (n 6) was significantly lower than the younger group (*P* = 0.02) (n 8)

We tested the TNFRSF4 protein levels by IHC and examined the protein expression in 14 melanoma samples where the normal control slides showed a negative detection of TNFRSF4 protein (Fig. 8, Supplementary Table 3). The TNFRSF4 expression was observed to be lower expressed in the older of melanoma tissues, and higher in the younger age group (*P* = 0.02) (Fig. 9).

## Discussion

People over the age of 65 who have survived cancer make up a large part of the general population, and the difference in survival rates between people who have cancer and those who don't has gotten more prominent in recent years [16]. Individuals aged 60 to 80 years old or older account for nearly half of all malignancies diagnosed today, and patients aged 60 to 80 account for approximately 80% and 30% of cancer-related fatalities, respectively [17]. As a result, immunosenescence has recently risen to the forefront of medical discussion, as a result of the development of novel immunomodulatory antibody therapies that rely on the patient's immune system to manage malignancies and that were first approved in MM patients, prompting an explosion of new research and debate. Our research aims to find the risk score genes that might be to blame for the age difference and the survival outlook.

The CKTTD database contains checkpoint protein targets, all of which have experimental evidence that has been confirmed and curated from 10 649 publications using an advanced text-mining method. In addition, it is a carefully managed resource relevant to available checkpoint targets and immunotherapy treatments for cancer. It is the first online resource of its sort, and the data in CKTTD is freely accessible to any scientist working in cancer immunotherapy and checkpoint molecule drug development. CKTTD offers users a concise overview of checkpoint targets and the treatment options available for checkpoint molecules or their regulatory pathways, backed up by verified data. CKTTD will increase further our knowledge of the checkpoint and immunotherapy mechanisms underlying human cancer. Notably, CKTTD will serve as a valuable bioinformatics resource for developing checkpoint-targeted therapeutics. In recent years, it has been established that combining targeted medicines directed at the tumor microenvironment with traditional chemo- or radiotherapy may increase cancer patient efficacy in clinical practice. We look forward to other therapy options that target checkpoint molecules to enhance patient outcomes and ultimately defeat cancer [11, 18]. The majority of the results of aged and MM patients, whether in a localized or remote location and in the ICI and non-ICI eras, are still controversial [19, 20]. However, almost the older MM patients exhibit more adverse and poorer prognosis than younger patients [21–25]. Overall, our cohort study reported that older people experience a worse prognosis than younger patients. We reported a significant novel, unique risk score for genes that was applied from the recent ICI genes database, CKTTD. This score will help with prediction and risk identification for MM patients. The aging effect was clear in this cohort in general and was significantly worse for the adults than for the non-adults. Further, we looked at the risk genes, correlated them with aging, and found that oncosupressor genes that are less common in older MM patients than in younger MM patients.

In addition to CSF1, TLR8, TLR4, and NOX4, TNFRSF4 was the most significantly associated and adversely correlated with age in both TCGA and GEO with aging. This risk score signature may play an essential role in the future as targeted ICI genes increase its expression for adult people in the immunotherapy era. The literature is fairly reported for this new gene about TNFRSF4 and its clinical efficacy and /or associated immune cells. In leukemic stem cells, stimulation of TNFRSF4-signaling did not deplete Tregs but impaired the potential of Tregs to protect LSCs from CD8 + CTL-mediated death. In the bone marrow of newly diagnosed chronic myeloid leukemia patients, TNFRSF4 mRNA levels were dramatically raised and linked with the expression of the Treg-restricted transcription factor FOXP3 [26]. Survival and TNFRSF4 expression with different immune cells were investigated in our study. The survival prediction of TNFRSF4 and associated age was significantly presented as a novel prognostic model. In the experiments, we tested TNFRSF4proten expression in 14 patients and investigated the score of the expression, which revealed a significant increase in the younger group of patients (< 65) compared to the older group. The patients' data, on the other hand, were small, but the significant difference was clear. Finally, our research identifies specific genes and gene pathways that may be responsible for the aging-related decrease in life expectancy. Also, we think about what might be behind these changes and what might be interesting research subjects for future studies on older people.

It is necessary to consider the limitations of this investigation. Firstly, owing to the study's retrospective character, it was difficult to rule out confounding variables that may have influenced the patients' prognosis in the future. Secondly, we only selected target data from TCGA and GEO public databases through biological algorithm approaches. Thirdly, though the experimental work validation was reported, further investigation needed to be done by different methods. Moreover, it is relatively weak to consider only mRNA expression and protein expression because many more complicated mechanisms contribute to MM. Finally, further functional research is needed to find out how prognostic genes in MM work together and how they affect both prognosis and treatment.

In summary, this study aimed to determine the predictive value of a novel ICI gene-related risk score derived from a new ICI gene list in melanoma patients. Additionally, we identified a novel ICI gene (TNFRSF4), a tumor suppressor gene associated with age-related survival inequalities related to immune cell infiltration, which may be related to the development of aggressive behavior and carcinogenesis in adult patients. To identify potential therapeutic targets, extensive research and large cohort data are required to understand how the gene's products function as well as how melanoma patients react as they get older.

## Supplementary Information

**Additional file 1:** **Supplementary Table 1.** Characteristics of malignant melanoma patients.

**Additional file 2:** **Supplementary Table 2. **CKTTD ICI genes: GO and KEGG pathways.

**Additional file 3:** **Supplementary Table 3.** Characteristics of affiliated hospital for malignant melanoma patients.

**Additional file 4:** **Supplementary Figure 1**: univariate hazard model and multivariate hazard model regression A. 40 genes with a significant difference in low vs. high expression (*P*= <0.05) resulted B. The resulted 6 genes with low vs. high expression as risk score genes.

**Additional file 5:** **Supplementary Figure 2.** A. overall survival rate for patients in the high-risk group was considerably lower than the rate for patients in the low-risk group in the risks core. (*P* = < 0.0001) B. the 6-gene signature's predictive potential was evaluated using a nomogram.

**Additional file 6:** **Supplementary  Figure 3.** The risk score genes as oncosuppressors (their high expression was related with greater survival than their low expression) with selected top 6 genes (*P*=< 0.05).

**Additional file 7:** **Supplementary  Figure 4.** ICI genes were illustrated and described in the context of the PPI network for CKTTD.

**Additional file 8:** **Supplementary Figure 5.** Spearman's rho r  value and statistical significance of the risk score and immune cells.

## Data Availability

The datasets generated and/or analysed during the current study are available in the GDC portal repository, [https://portal.gdc.cancer.gov/].
