# Supplementary material for: Immune checkpoint inhibitor (ICI) genes and aging in malignant melanoma patients: a clinicogenomic TCGA study
Source: BMC Cancer. 2022 Sep 13;22:978. doi: 10.1186/s12885-022-09860-2 (PMC9469583; doi:10.1186/s12885-022-09860-2)
Supplement: Supplementary file 1 — Additional file 1: Supplementary Table 1. Characteristics of malignant melanoma patients. [file 12885_2022_9860_MOESM1_ESM.docx]

| **Parameters** | **N=414(%)** | | |
| --- | --- | --- | --- |
| **Survival status** |  |  |  |
| **Alive** | 228(55.1) |  |  |
| **Dead** | 186(44.9) |  |  |
| **Age** |  |  |  |
| **20- 65** | 267(64.5) |  |  |
| **>65** | 147(35.5) |  |  |
| **Gender** |  |  |  |
| **Male** | 260(62.8) |  |  |
| **Female** | 154(37.2) |  |  |
| **Stage** |  |  |  |
| **0-II** | 228(55.1) |  |  |
| **III-IV** | 186(44.9) |  |  |
| **RISK** |  |  |  |
| **high** | 207（50.0） |  |  |
| **low** | 207（50.0） |  |  |

Table 1 Characteristics of malignant melanoma patients
