# Supplementary material for: Immune checkpoint inhibitor (ICI) genes and aging in malignant melanoma patients: a clinicogenomic TCGA study
Source: BMC Cancer. 2022 Sep 13;22:978. doi: 10.1186/s12885-022-09860-2 (PMC9469583; doi:10.1186/s12885-022-09860-2)
Supplement: Supplementary file 2 — Additional file 2: Supplementary Table 2. CKTTD ICI genes: GO and KEGG pathways. [file 12885_2022_9860_MOESM2_ESM.docx]

**Supplementary table 2**

| source | term_name | adjusted_p_value |
| --- | --- | --- |
| GO:BP | aging | 0.01206731 |
| GO:BP | cell aging | 0.022802987 |
| GO:BP | immune system process | 1.13E-28 |
| GO:MF | protein kinase activity | 1.49E-10 |
| GO:MF | phosphotransferase activity, alcohol group as acceptor | 3.40E-09 |
| GO:MF | molecular transducer activity | 7.32E-09 |
| GO:CC | cell surface | 2.62E-17 |
| GO:CC | external side of plasma membrane | 1.75E-11 |
| GO:CC | side of membrane | 1.80E-09 |
| GO:CC | plasma membrane | 6.03E-09 |
| KEGG | Cytokine-cytokine receptor interaction | 2.47916E-05 |
| KEGG | Cell adhesion molecules | 0.004146884 |
| KEGG | Toll-like receptor signaling pathway | 0.026239212 |
| KEGG | p53 signaling pathway | 0.042450347 |

**CKTTD ICI genes: GO and KEGG pathways**
