# Supplementary material for: Immune checkpoint inhibitor (ICI) genes and aging in malignant melanoma patients: a clinicogenomic TCGA study
Source: BMC Cancer. 2022 Sep 13;22:978. doi: 10.1186/s12885-022-09860-2 (PMC9469583; doi:10.1186/s12885-022-09860-2)
Supplement: Supplementary file 3 — Additional file 3: Supplementary Table 3. Characteristics of affiliated hospital for malignant melanoma patients. [file 12885_2022_9860_MOESM3_ESM.docx]

| **Parameters** | **N=14(%)** | | |
| --- | --- | --- | --- |
| **Survival status** |  |  |  |
| **Alive** | 7(50) |  |  |
| **Dead** | 7(50) |  |  |
| **Age** |  |  |  |
| **< 65** | 8(57) |  |  |
| **>=65** | 6(43) |  |  |
| **Gender** |  |  |  |
| **Male** | 8(57) |  |  |
| **Female** | 6(43) |  |  |
| **Stage** |  |  |  |
| **II-III** | 5(35) |  |  |
| **IV** | 9(65) |  |  |

Table 3 Characteristics of affiliated hospital for malignant melanoma patients
