# Supplementary figures and images for: Immune checkpoint inhibitor (ICI) genes and aging in malignant melanoma patients: a clinicogenomic TCGA study
Source: BMC Cancer. 2022 Sep 13;22:978. doi: 10.1186/s12885-022-09860-2 (PMC9469583; doi:10.1186/s12885-022-09860-2)

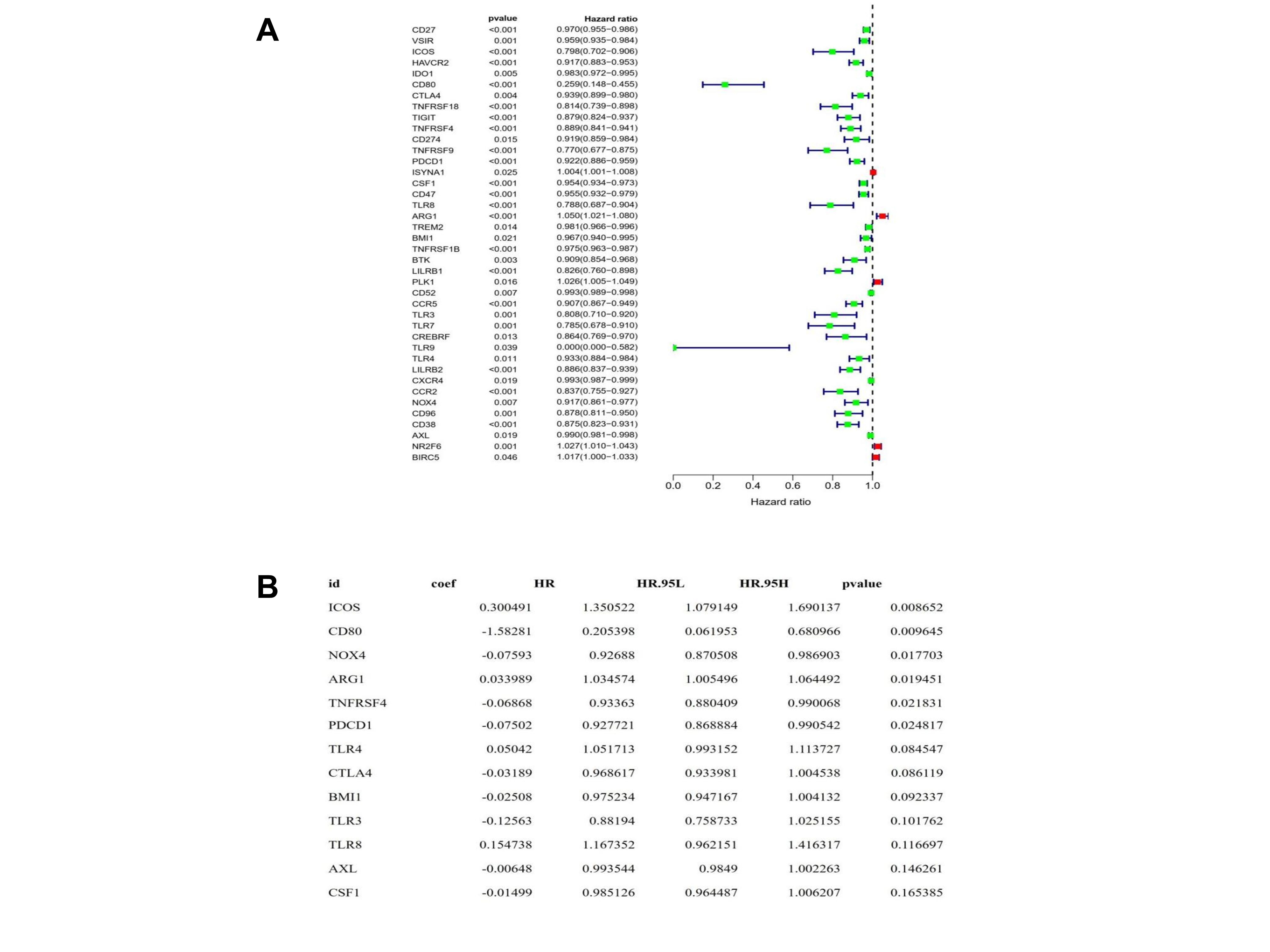

Supplement: Supplementary file 4 — Additional file 4: Supplementary Figure 1: univariate hazard model and multivariate hazard model regression A. 40 genes with a significant difference in low vs. high expression (P= <0.05) resulted B. The resulted 6 genes with low vs. high expression as risk score genes. [file 12885_2022_9860_MOESM4_ESM.tif]

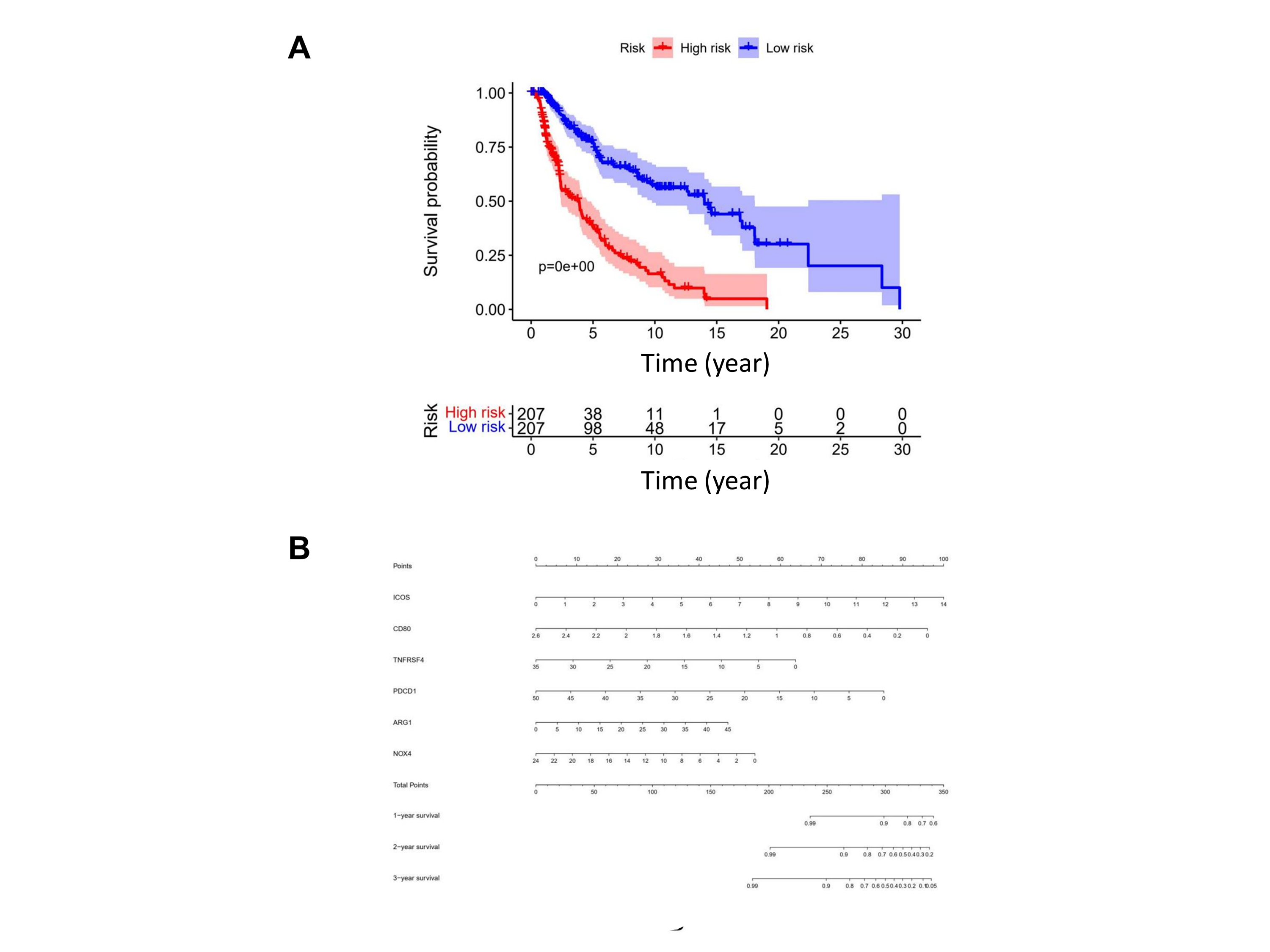

Supplement: Supplementary file 5 — Additional file 5: Supplementary Figure 2. A. overall survival rate for patients in the high-risk group was considerably lower than the rate for patients in the low-risk group in the risks core. (P = < 0.0001) B. the 6-gene signature's predictive potential was evaluated using a nomogram. [file 12885_2022_9860_MOESM5_ESM.tif]

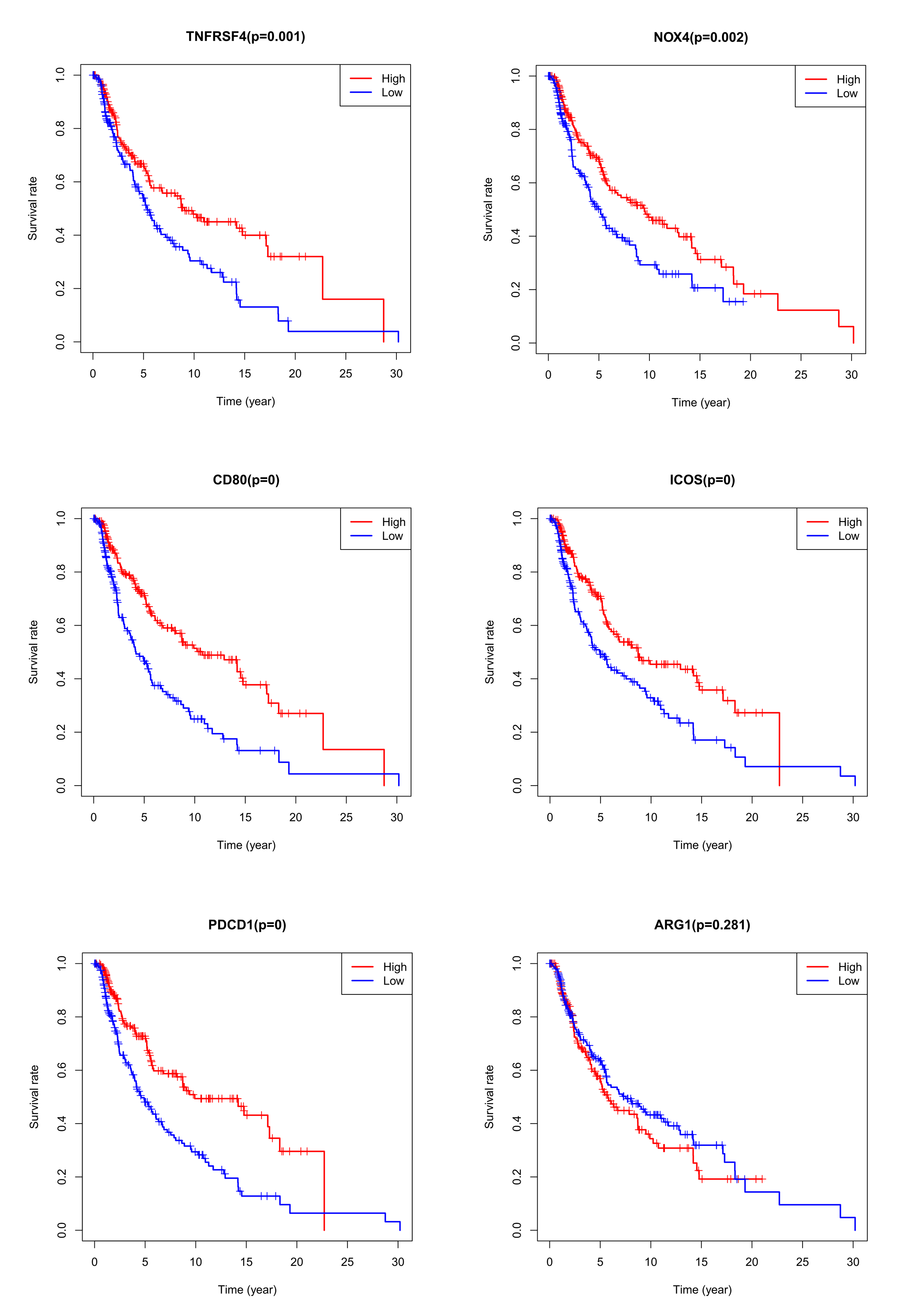

Supplement: Supplementary file 6 — Additional file 6: Supplementary Figure 3. The risk score genes as oncosuppressors (their high expression was related with greater survival than their low expression) with selected top 6 genes (P=< 0.05). [file 12885_2022_9860_MOESM6_ESM.tif]

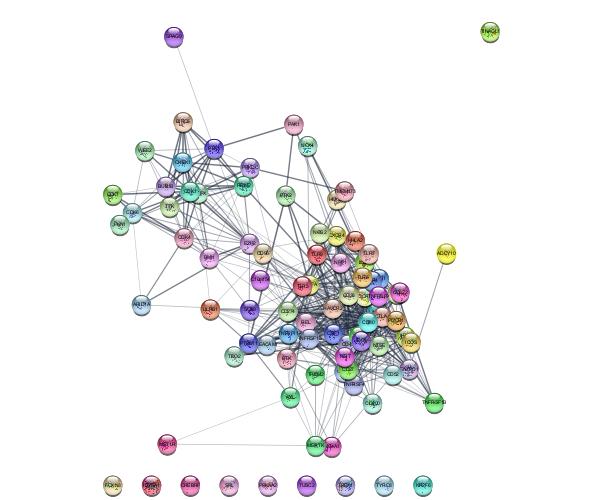

Supplement: Supplementary file 7 — Additional file 7: Supplementary Figure 4. ICI genes were illustrated and described in the context of the PPI network for CKTTD. [file 12885_2022_9860_MOESM7_ESM.jpeg]

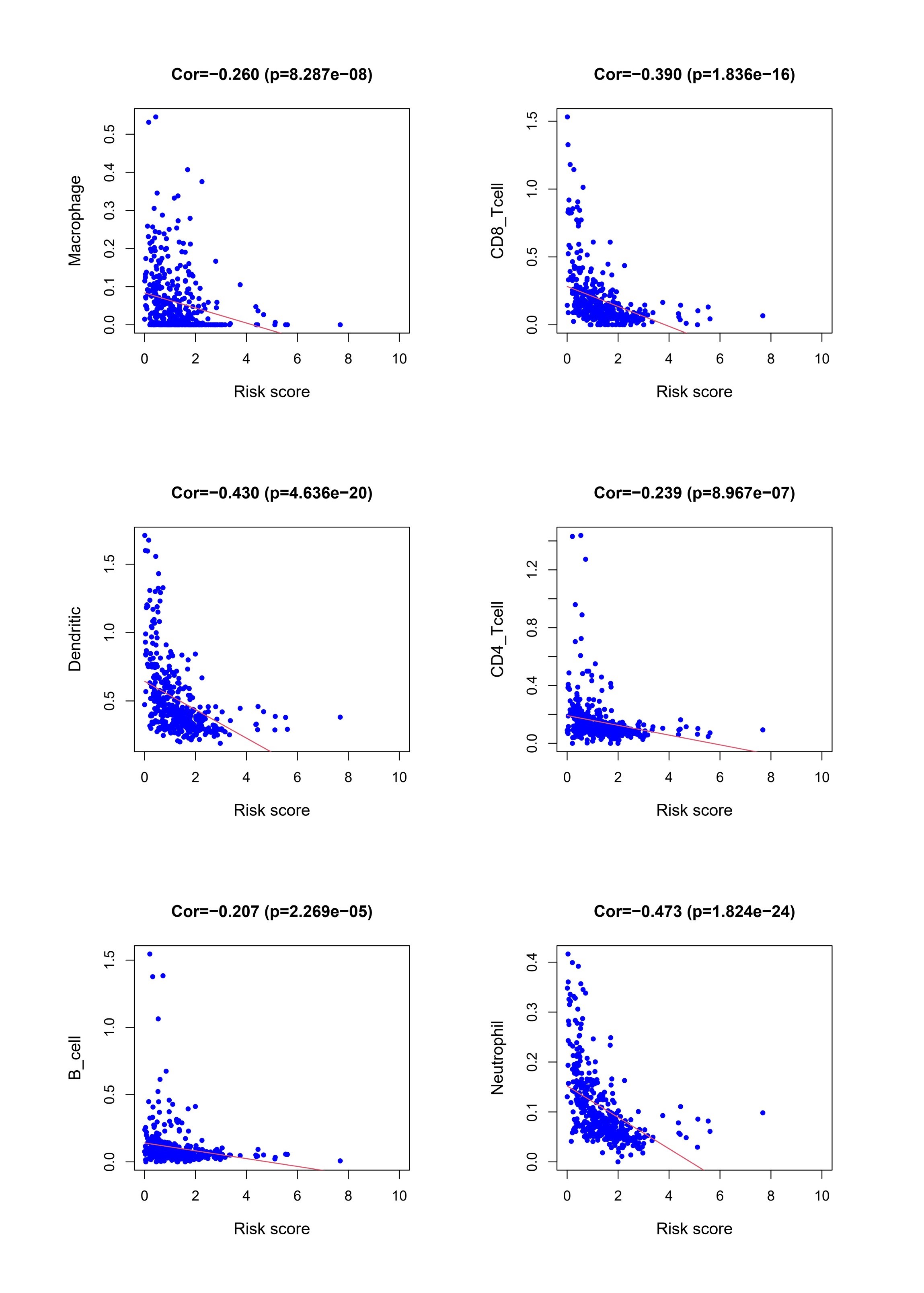

Supplement: Supplementary file 8 — Additional file 8: Supplementary Figure 5. Spearman's rho r value and statistical significance of the risk score and immune cells. [file 12885_2022_9860_MOESM8_ESM.jpg]
